# Enhancing Efficiency and User Experience of Digital Community Health Worker Payments in Zanzibar: Implementation Report

**DOI:** 10.2196/65325

**Published:** 2025-05-28

**Authors:** Lee Pyne-Mercier, Krishna Jafa, Susan Maigua, Jennifer Muli, Elijah Gichinga, Antony Khaemba, Nitusima Kataraia, Aisha Mohammed, Frank Kamangadazi Tembo, Imran Esmail, Giulia V R Besana, Heiko Hornung, Ali Makame Zubeir

**Affiliations:** 1 University of Washington, 3980 15th Ave NE, Seattle, WA, 98105, United States, 1 206-696-8855; 2 Medic, San Francisco, CA, United States; 3 D-tree, Norwell, MA, United States; 4 Ministry of Health, Zanzibar, United Republic of Tanzania

**Keywords:** digital payment, mobile health, digital health, mobile payments, mobile money, user experience, Zanzibar, Africa, implementation report, community health worker

## Abstract

**Background:**

Community health workers (CHWs) are essential for achieving universal health coverage and reaching the Sustainable Development Goals. Paying CHWs for their work increases their motivation and effectiveness, and is recommended by the World Health Organization and advocacy organizations such as the Community Health Impact Coalition. Many implementing organizations currently pay CHWs using mobile money or other digital means. However, most payment systems are designed without the involvement of CHWs.

**Objective:**

In this implementation report, we describe efforts to improve efficiency, accuracy, and user experience of the CHW payment process of the Jamii ni Afya project in Zanzibar.

**Methods:**

We applied Medic’s design process to develop new functionality for the open-source Community Health Toolkit. We reviewed documentation and engaged with users to understand their needs and experiences with the current payment system. This information formed the basis of technical specifications, which were developed into a revised workflow. The workflow was iteratively tested and refined. Several steps that were managed offline, such as resolving payment discrepancies, were formalized and incorporated into the workflow. We conducted user acceptance testing to assess functionality and user experience.

**Implementation (Results):**

The workflow was able to accurately translate programmatic data into payment information for each CHW and securely transmitted this information to a payment service provider. User acceptance testing revealed that CHWs felt the revised payment system provided them with more information and gave them a greater sense of control. Program staff felt the workflow would increase the efficiency and accuracy of the payment process, while simplifying the resolution of payment discrepancies.

**Conclusions:**

Engaging users in the design and optimization of digital payment systems has the potential to improve the efficiency and accuracy of digital payment systems while enhancing satisfaction among all users, contributing to improved sustainability and impact of CHW programs. Definitive conclusions will depend on evaluation of the system after implementation.

## Introduction

### Context

In 2015, countries worldwide committed to the Sustainable Development Goals (SDGs). The SDGs set ambitious targets for the health workforce [1]. Most countries are not on track to meet the 2030 targets without additional investments [2]. The World Health Organization (WHO) has projected a shortage of 18 million health workers by 2030 if current trends continue [3].

Community health workers (CHWs) provide health promotion and preventive and curative care for people at their doorstep. CHWs can effectively deliver various interventions, including maternal and child health services, family planning, and prevention and management of infectious and noncommunicable diseases [4]. For example, in Rwanda and Liberia, CHWs treat half of malaria cases, and in many countries, they were essential in maintaining health services during the COVID-19 pandemic [5]. In Brazil, CHWs reached two-thirds of the population and have helped reduce child mortality by 75% [6]. CHWs are estimated to provide a return on investment of US $10 for every US $1 invested [7].

Most CHWs in low- and middle-income countries (LMICs) remain unpaid despite evidence that CHWs save lives and are highly cost-effective [4,8-10]. In Africa, 86% of CHWs are volunteers with little or no compensation [11]. The WHO’s 2018 CHW guidelines recommend remuneration commensurate with job demands, complexity, hours worked, and training [9]. Satisfaction with incentives is shown to improve performance while reducing attrition [12,13]. In contrast, inadequate, partial, or delayed payments reduce motivation and intervention coverage [13].

Significant challenges have been documented with cash payments for CHWs. The logistics needed to safely transport and accurately disburse cash become increasingly difficult with scale. This can result in delayed and incomplete payments, corruption, high administrative costs, and security risks [13-18]. Digital payments have been championed as a solution. According to researchers at Makerere University, “digital payments can create a transparent record that can improve management and reduce payment delays and leakage of funds. Digitizing payments can increase the likelihood of health workers receiving complete, on-time, and convenient payment, increase transparency, reduce resource leakage, and improve service delivery’s cost-effectiveness” (Makerere University, 2022; website changed and material no longer available).

### Digital CHW Payments

D-tree was among the first organizations working in an LMIC to adopt digital payments. In 2011, D-tree piloted mobile money payments for CHWs, supervisors, and emergency transportation services [19,20]. In the next decade, organizations introduced digital payments for CHWs, such as malaria spray teams in Zambia, CHWs in Bangladesh and Kenya, tuberculosis screeners in Pakistan, and many others [19,21-24].

Donors accelerated the digital transition. In 2014, the US Agency for International Development mandated digital payments as the default payment mode, including those for CHWs [25]. The Global Polio Eradication Initiative began scaling up digital payments for vaccination campaign workers in 2020, reaching >250,000 recipients within a year [13,26]. The uptake of digital payments for health workers has been rapid compared to an average of 14 years for drug and/or technology innovations to reach scale [27].

Because most reports on digital payments appear in the gray literature, they tend to present positive perspectives, overlooking challenges and limitations. Regardless, several challenges have been identified. Health worker registries are often poorly maintained [13]. Resolving payment discrepancies can be time-consuming [28,29]. There are gaps in phone ownership, cellular networks, and mobile money agents [13,15,19,28]. Some CHWs have low literacy, are unfamiliar with technology, or distrust mobile money [19,28]. Requirements for a national identity document can be a barrier [19]. Financial management capacity may be insufficient [13]. Finally, transaction fees can significantly reduce the take-home pay of recipients [13,30].

Despite the rapid increase in digital payments for CHWs, there is limited evidence on the topic. There is a need for more research on the effect of digital payments on health workers’ morale, performance, quality of care, intervention coverage, and health outcomes [14,17,18]. Thankfully, there are new initiatives aiming to fill the gap, notably the Digital Health Payment Initiative at Makerere University [14,31].

### Participating Entities

Medic serves as the steward of the Community Health Toolkit (CHT), an open-source digital public good that supports health workers as they deliver care [32]. The CHT is a collection of technologies and resources that enable implementers to support various features and health issues. As of December 2023, in total, 75,241 CHWs and their supervisors in 18 countries used CHT-based tools to deliver care during 117.5 million home visits [33].

Medic has embraced human-centered design, engaging individuals who will use the tools in the environments where they are deployed. The CHT is intended to empower health workers to care for members of their community, and Medic’s design process considers the interests of the person overall [34]. Medic has intentionally situated its staff in the communities where our tools are deployed, including Kenya, Nepal, Senegal, and Uganda.

D-tree is a global health organization working to improve access to quality health care. In Zanzibar, D-tree works with the Ministry of Health (MOH) to support Jamii ni Afya (JnA), one of the world’s first nationally scaled, digitally enabled community health programs. JnA includes about 2300 CHWs and 220 CHW supervisors serving a population of nearly 2 million. The JnA smartphone app (built on CHT) guides CHWs to provide services for maternal and child health, nutrition, water, sanitation, hygiene, and early childhood development.

To support, incentivize, and monitor CHWs, a pay-for-performance scheme is used to calculate monthly remuneration. The current maximum monthly payment is approximately US $21 for CHWs and US $13 for supervisors (who are generally facility-based MOH staff). The program is included in Zanzibar’s digital and community health strategies [35,36]. Higher rates of facility deliveries and reduced child stunting have been documented in the 5 years following JnA’s scale-up [35].

### Problem Statement

While JnA already made mobile money payments to CHWs, the process had several drawbacks. Multiple steps were needed to collect performance data, review them, and translate them into digital payments. The CHT and digital payment systems were not integrated. Users felt that they did not always have the information they needed, and several routine tasks were managed offline. In addition, financial data were not yet included in CHT. We are not aware of peer-reviewed literature documenting how to proactively involve CHWs in designing digital payment systems. This implementation report documents our experiences working on CHW digital payments in Zanzibar and follows the i-CHECK-DH (Guidelines and Checklist for the Reporting on Digital Health Implementations) guidelines (Checklist 1) for reporting on digital health implementations [37].

### Aims and Objectives

The goal of this initiative was to improve the efficiency, accuracy, and user experience of the CHW payment process. Specific objectives included reducing effort to make payments, increasing accuracy, providing real-time information to all users, and streamlining discrepancy resolution. This implementation report describes the process to design the digital tool in the preimplementation phase through the lens of user acceptance testing (UAT). UAT evaluated the product’s user experience, functionality, and interoperability with a small number of users. It is outside of the scope of this report to measure more distal outcomes, such as coverage, accuracy, or impact. Once the digital payments solution is implemented, we will be positioned to report on these outcomes.

## Methods

### Blueprint Summary

Medic designers started by gathering information on current processes and systems via reviewing existing documentation and calls with JnA project staff. In March 2023, a team of 3 Medic staff with design and software development expertise traveled to Zanzibar for a design visit. In addition to gathering information about the current payment system, we aimed to understand users’ experience with the system. We conducted interviews and focus groups with program staff and CHWs. Participants’ age ranged 20-50 years. We were also able to shadow supervisors and attend monthly meetings that were an important step in the payment process.

**Figure 1.**
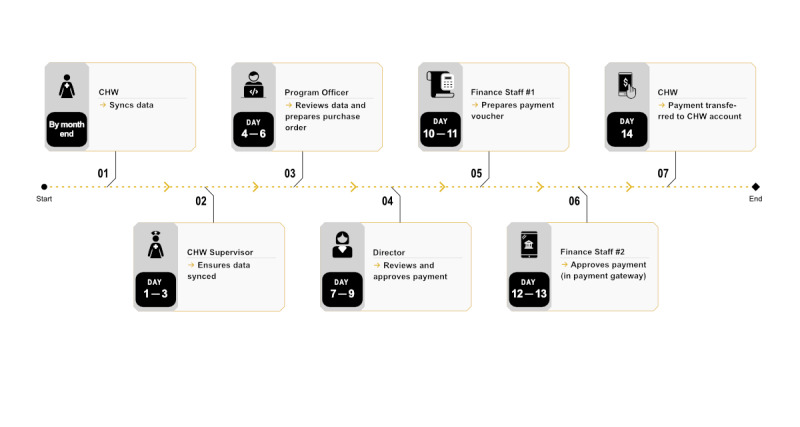
Existing payment process map. CHW: community health worker.

D-tree’s existing payment process is shown in Figure 1. The entire process takes approximately 2 weeks once data are synced. CHWs have 5 days at the end of each month to sync their data. Supervisors ensure that data are synced for all CHWs. Program officers subsequently review data using a custom-built dashboard. Data are exported to Microsoft Excel to calculate payments. Once approved by the project director, the finance team reformats data per payment service provider requirements, and this is uploaded using a web-based portal. Payment discrepancies are handled case-by-case by program staff.

Challenges identified by users were one input for the design. Poor connectivity and depleted mobile data often prevented CHWs from syncing, leading to payment discrepancies. CHWs felt uncertain when to expect payments or how calculations were made. Resolving payment discrepancies was time-consuming, and CHWs would sometimes incur out-of-pocket costs to follow up on payments. Some supervisors adopted improvised solutions such as the use of screenshots from CHWs’ phones to verify data accuracy.

Finance staff explained that manual steps sometimes led to errors. The existing payment service provider did not offer an easy, robust, and customizable way to integrate with CHT. D-tree is transitioning management for JnA to the Zanzibar MOH by 2026, and the payment scheme needs to integrate with government administrative systems. The workflow needed to be sustainable by the program without external inputs and feasible within CHT’s parameters while creating value for users.

We validated findings with stakeholders via videoconference. Part of our process entailed creating personas who interacted with the software. For each persona, we considered their job responsibilities, needs, context, and motivations. Personas were based on information collected from the design visit and included CHWs, supervisors, monitoring and evaluation officers, finance officers, directors, and district health staff.

We developed a draft workflow and refined it during several remote coworking sessions. Mostly, the steps in the workflow did not change from the existing process. However, several steps that used text message or phone communication were formalized and incorporated into the workflow. We identified 7 candidate Payment Service Providers (PSPs) through web searches and consultation with local informants. Selection criteria were adapted from global best practice [38]. The PSP was selected because of their presence in Zanzibar, available digital payment modalities (mobile networks and banks), data security, existing application programming interfaces (APIs), and reputation.

**Figure 2.**
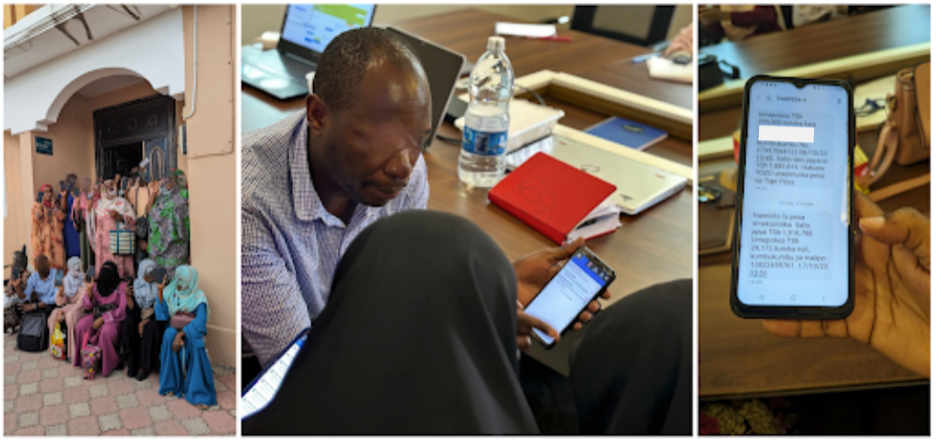
User acceptance testing conducted in Zanzibar.

**Figure 3.**
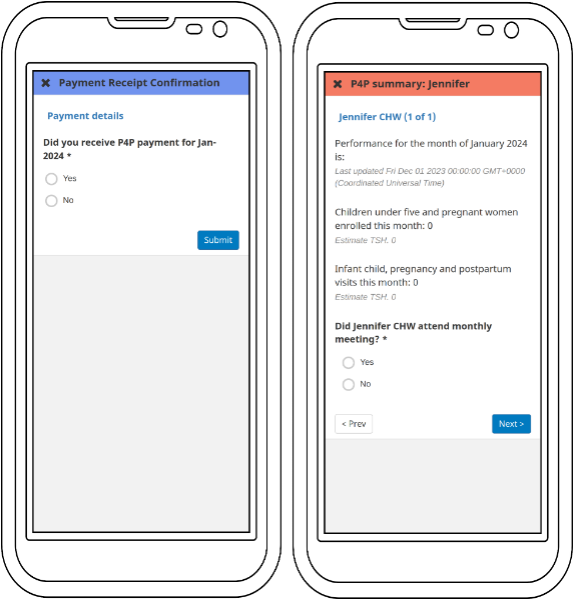
Screenshots of 2 example forms in the CHT (Community Health Toolkit) digital payments workflow. CHW: community health worker.

We tested the workflow in several stages using a structured test plan and we incorporated feedback iteratively. By October 2023, we had finalized integration with the PSP and returned to Zanzibar for 4 days of user acceptance testing (Figure 2). Our goals with UAT were to assess functionality of the software and to gather user feedback. Medic’s designers observed users interacting with the system and interviewed them about their experiences. Participants included 23 CHWs, 8 supervisors, 8 MOH staff, and 10 D-tree staff. Participants were selected through a purposive random sample and included CHWs from both urban and rural settings because we hypothesized they might have different challenges. Examples of the user interface are shown in Figures3  4. Screenshots show the English translations, but the UAT was conducted in Kiswahili. Personally identifiable information in the screenshots have been replaced by test data. Results from the UAT will inform changes to the software before finalization.

**Figure 4.**
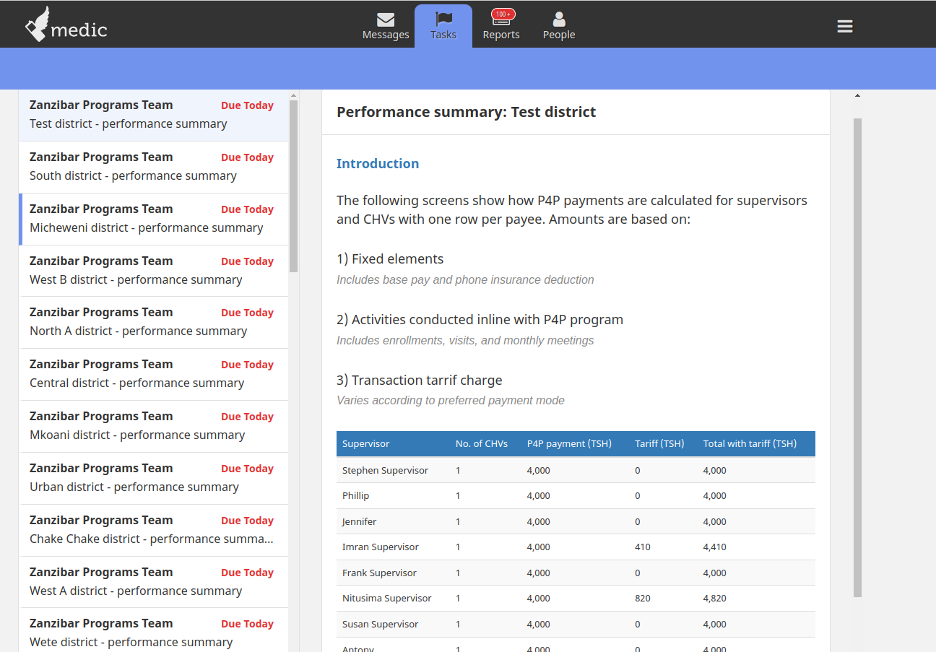
Screenshot of an example digital payments summary. CHV: community health volunteer.

### Technical Design

The workflow was built using the CHT, which serves as the backbone of the JnA app [39]. CHT was selected by the government of Zanzibar because it is open-source, has a growing support community, can be hosted locally, and the expertise to manage the software is readily available [35]. The software was designed to function in low-bandwidth environments using affordable Android smartphones. CHWs and supervisors generally access JnA using smartphones, while office staff access JnA using a web browser.

### Target Audience

The target audience consisted of all JnA stakeholders who were involved in the payment process, including CHWs, supervisors, and staff at the MOH and D-tree. The broader CHT community is a secondary audience. As an open-source tool, CHT features can be widely adapted and used by community members.

### Data

The workflow we developed uses existing data, including personal identifiers for CHWs, process data, and mobile banking details. Data are collected during enrollment and while CHWs and supervisors conduct their routine activities. The workflow hierarchy was designed to protect sensitive information and is governed by a data privacy policy, which users consent to during enrollment [40]. Financial data are managed in compliance with government regulations [41]. We plan to conduct penetration testing to ensure data security at a later stage of development.

### Interoperability and Integration

CHT can exchange data with other components of national health information systems based on standards including Fast Healthcare Interoperability Resources, Open Health Information Exchange, and the Open Health Information Mediator [39]. We built a custom mediator to calculate performance-based payments and to disburse them via the payment service provider’s API. Available standards for financial service interoperability do not include health use cases [42]. There is a need for interoperability standards in health-related financial services.

### Budget Planning and Sustainability

The Community Health Toolkit is free to use but requires funding for adaptation, hardware, and hosting. These costs are included in the Zanzibar Community Health Strategy, in addition to CHW stipends, commodities, training, and logistics costs [35]. JnA’s annual operating cost is estimated at US $2.73 million per year or US $1.70 per capita. The government is currently paying the salaries of CHW supervisors and has committed to fully fund the program by 2026 with the vision of absorbing CHW as part of the health workforce. A financing strategy is currently under development. Payment service provider fees were between US $0.30 and US $0.50 per transaction during testing, representing a significant percentage of the total payments. However, fees are negotiable, and our experience was based on a very small transaction volume during the test phase.

## Implementation (Results)

We assessed the product’s functionality, user experience, and interoperability with 49 users. The workflow functioned as designed and was positively received by staff and CHWs. Most steps felt familiar, since additional functions were generally incorporated into existing forms. Participants recommended several improvements that would simplify data entry, remove unnecessary information, enable detailed or summary views, and highlight outlier data. They also recommended a process for escalating approvals to avoid delays if approvers were not available.

CHWs reported feeling a greater sense of control because they confirm their data at the beginning of the payment cycle and report payment receipt at the end. They appreciated seeing how their stipends are calculated in the app. While increased transparency is thought to increase motivation and trust, there could be other consequences such as task prioritization based on reimbursement levels. It will be important to monitor changes over time.

Supervisors felt that the workflow would facilitate performance management by providing accurate, timely data. Program staff felt that the software reduced their workload, particularly less enjoyable tasks such as reformatting data to meet PSP requirements. They thought that automated calculations would increase accuracy and believed that the workflow’s ability to document approvals was easier than existing processes. Several user groups felt that the process for tracking and resolving payment discrepancies would increase responsiveness.

**Figure 5.**
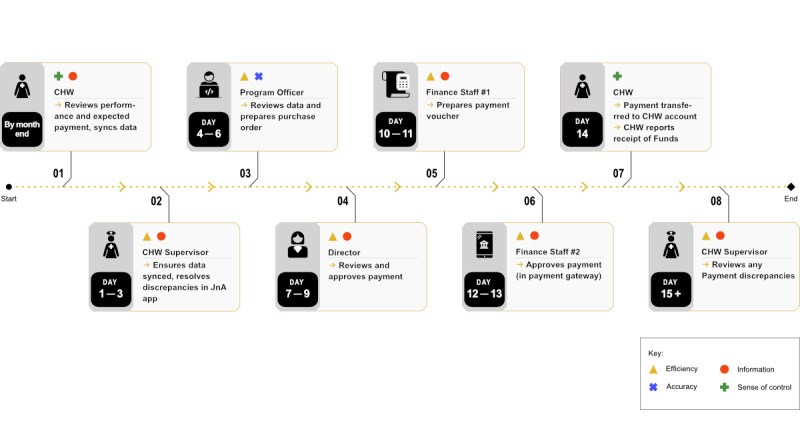
Simplified monthly workflow with perceived benefits at each step. CHW: community health worker.

CHT has been primarily used for clinical and programmatic data, and its ability to manage financial information was unproven. The software reliably used performance data to calculate payments and securely communicated this information with the PSP. Some of the developed functionality, such as display of tabular information, is based on the XForm standard and can be deployed on any XForm-compatible platform like OpenDataKit to empower effective decision-making. A simplified visual of the steps in the workflow and the perceived benefits at each stage are shown in Figure 5.

## Discussion

### Principal Findings

We learned several lessons from this work. It was feasible to involve CHWs in redesigning the JnA payment system. However, their engagement was limited to the design visit and UAT. We could have included CHWs as part of the team throughout the development process for deeper involvement. Remote coworking sessions were sometimes challenging because they are generally shorter and less interactive than in-person design meetings. We could have accelerated implementation with more in-person collaboration. While Medic’s Nairobi-based team were generally familiar with JnA’s infrastructure and Zanzibar’s health system, we could have improved collaboration by forming a unified team comprising members from both organizations. CHT could manage financial, health, and programmatic data, leading to efficiencies. Key lessons are summarized in Textbox 1.

CHWs are an important tool to promote health equity by increasing health care access to households that face financial, geographic, or other barriers to accessing care [43]. This implementation report contributes to efforts to professionalize the community health workforce. Professionalized CHWs reportedly have greater health impacts than unpaid volunteer CHWs [44]. Paying CHWs can also reduce gender and income inequality because most CHWs are low-income women. Payment enables CHWs to address their own livelihood needs while becoming trusted role models in the community [8,13].

Engaging users in designing digital payment systems is the exception rather than the rule. Many current payment systems were designed without CHW involvement and do not meet their needs [29]. During testing, CHWs reported feeling a greater sense of control, which reportedly reduces stress and improves health [45]. Increased transparency and accountability between the program and CHWs can allow them to plan how much time to devote to CHW duties or to other income-generating activities, assuring a predictable income. We believe that co-designing payment systems with payees should become the norm.

Beyond digital payment for CHWs, mobile money integration with CHT is an opportunity to accelerate innovation. D-tree has a long history of mobile money innovation, and this integration could facilitate additional use cases, such as birth preparedness savings, community health insurance, emergency transport payments, cash transfers, savings groups, and many others that will only become apparent with time.

Textbox 1. **Key lessons.**

- It is feasible to engage community health workers (CHWs) in the design of digital payment systems, leading to better alignment with users’ needs. Engaging users at key decision points is helpful, but including them in the entire process may lead to more transformative designs.
- Remote coworking has limitations, so budgeting for in-person time may improve communication and accelerate development timelines.
- Community health information systems can manage financial data in addition to clinical and programmatic data, which may lead to efficiencies and new use cases.

### Conclusions

The question is no longer about the feasibility of digital payments for CHWs, but how to do it efficiently, in a way that best meets the needs of all users. We showed that the open-source CHT could manage financial data and meet various user needs. We demonstrated the utility of engaging users in designing a digital payments system. More robust evaluation is planned as part of the next phase of development, including the impact of the workflow on timelines, workload, accuracy, motivation, and programmatic outcomes.

## Supplementary material

10.2196/65325 Checklist 1 i-CHECK-DH (Guidelines and Checklist for the Reporting on Digital Health Implementations) checklist.

## Abbreviations:

API: application program interface
CHT: Community Health Toolkit
CHW: community health worker
HCD: Human Centered Design
i-CHECK-DH: Guidelines and Checklist for the Reporting on Digital Health Implementations
JnA: Jamii ni Afya
LMIC: low- and middle-income country
MOH: Ministry of Health
PSP: Payment Service Provider
SDG: Sustainable Development Goal
UAT: User Acceptance Testing
WHO: World Health Organization
